# Fucoidan Prevents RANKL-Stimulated Osteoclastogenesis and LPS-Induced Inflammatory Bone Loss via Regulation of Akt/GSK3β/PTEN/NFATc1 Signaling Pathway and Calcineurin Activity

**DOI:** 10.3390/md17060345

**Published:** 2019-06-10

**Authors:** Sheng-Hua Lu, Yi-Jan Hsia, Kuang-Chung Shih, Tz-Chong Chou

**Affiliations:** 1 Graduate Institute of Life Sciences, National Defense Medical Center, Taipei 114, Taiwan; shooter741109512@gmail.com; 2 Dental Department and Devision of Oral and Maxillofacial Surgery, Taipei Tzu Chi Hospital, Buddhist Tzu Chi Medical Foundation, New Taipei City 23142, Taiwan; yjhsia@yahoo.com.tw; 3 Division of Endocrinology and Metabolism, Department of Internal Medicine, Tri-Service General Hospital, National Defense Medical Center, Taipei 114, Taiwan; shihkc19610909@gmail.com; 4 Division of Endocrinology and Metabolism, Department of Internal Medicine, Cheng-Hsin General Hospital, Taipei 112, Taiwan; 5 Department of Biotechnology, Asia University, Taichung 413, Taiwan; 6 China Medical University Hospital, China Medical University, Taichung 400, Taiwan; 7 Graduate Institute of Medical Sciences, National Defense Medical Center, Taipei 114, Taiwan; 8 Department of Pharmacology, National Defense Medical Center, Taipei 114, Taiwan

**Keywords:** fucoidan, RANKL, osteoclastogenesis, calcineurin, lipopolysaccharide, bone loss

## Abstract

Excessive osteoclast differentiation and/or function plays a pivotal role in the pathogenesis of bone diseases such as osteoporosis and rheumatoid arthritis. Here, we examined whether fucoidan, a sulfated polysaccharide present in brown algae, attenuates receptor activator of nuclear factor-κB ligand (RANKL)-stimulated osteoclastogenesis in vitro and lipopolysaccharide (LPS)-induced bone resorption in vivo, and investigated the molecular mechanisms involved. Our results indicated that fucoidan significantly inhibited osteoclast differentiation in RANKL-stimulated macrophages and the bone resorbing activity of osteoclasts. The effects of fucoidan may be mediated by regulation of Akt/GSK3β/PTEN signaling and suppression of the increase in intracellular Ca^2+^ level and calcineurin activity, thereby inhibiting the translocation of nuclear factor-activated T cells c1 (NFATc1) into the nucleus. However, fucoidan-mediated NFATc1 inactivation was greatly reversed by kenpaullone, a GSK3β inhibitor. In addition, using microcomputer tomography (micro-CT) scanning and bone histomorphometry, we found that fucoidan treatment markedly prevented LPS-induced bone erosion in mice. Collectively, we demonstrated that fucoidan was capable of inhibiting osteoclast differentiation and inflammatory bone loss, which may be modulated by regulation of Akt/GSK3β/PTEN/NFATc1 and Ca^2+^/calcineurin signaling cascades. These findings suggest that fucoidan may be a potential agent for the treatment of osteoclast-related bone diseases.

## 1. Introduction 

Bone homeostasis is largely controlled by the balance of the actions of bone-resorbing osteoclasts and bone-forming osteoblasts. Activation of osteoclasts and/or dysfunction of osteoblasts results in the development of various bone disorders such as osteoporosis and rheumatoid arthritis [1]. Receptor activator of nuclear factor-κB (RANKL) has been regarded as an important stimulator for osteoclast differentiation from the monocytes/macrophage lineage [2]. After the binding of RANKL to RANK receptor, many transcription factors such as c-fos, NF-κB, and nuclear factor-activated T cells c1 (NFATc1) are activated [3]. Several studies have indicated that NFATc1, in particular, plays an essential role in osteoclast differentiation and functions via upregulation of osteoclast-related genes, including cathepsin K, tartrate-resistant acid phosphatase (TRAP), and matrix metalloproteinase-9 (MMP-9) [4]. As expected, impaired osteoclastogenesis occurs in conditional NFATc1-deficient mice, resulting in osteopetrosis [5]. 

It is known that RANKL-induced osteoclast differentiation is a complex process that is modulated by multiple pathways [6]. Following stimulation with RANKL, the intracellular level of Ca^2+^ ([Ca^2+^]i) increases, markedly. Subsequently, calcium binds to calmodulin leading to activation of calcineurin, a ubiquitous serine-threonine phosphatase, which dephosphorylates the serine residues in NFATc1 and, in turn, enhances nuclear translocation of NFATc1 [7]. Conversely, RANKL-induced osteoclast differentiation was greatly inhibited by the inhibitors of calcineurin such as FK506 and cyclosporine A [8], suggesting that suppression of the Ca^2+^/calmodulin/calcineurin/NFATc1 axis is an effective strategy to attenuate RANKL-induced osteoclastogenesis. Glycogen synthase kinase 3 (GSK3), a serine/threonine kinase, has several biological functions, including regulation of metabolism, transcription, translation, cell growth, and apoptosis [9]. Notably, the GSK3β isoform is capable of phosphorylating NFATc1, resulting in suppression of NFATc1nucleotranslocation and enhancement of nuclear export of NFATc1, ultimately inhibiting osteoclast differentiation [10]. However, Akt-mediated GSK3β phosphorylation inactivates GSK3β, thereby promoting nuclear accumulation of NFATc1 and osteoclast differentiation [11,12]. Phosphatase and tensin homolog (PTEN) have been reported to inhibit osteoclastogenesis by suppressing phosphoinositide 3-kinase (PI3K)/Akt signaling via dephosphorylation of PIP3 to PIP2 [13]. In resting cells, GSK3β-mediated phosphorylation of PTEN at Thr 366 site activates PTEN, and therefore inhibits the PI3K/Akt pathway. However, under RANKL stimulation, the PI3K/Akt cascade is activated, leading to inactivation of GSK3β and PTEN, leading to osteoclastogenesis [13]. These findings highlight the importance of Akt/GSK3β/PTEN/NFATc1 and calcium/calcineurin/NFATc1 signaling pathways in the regulation of RANKL-induced osteoclast differentiation. 

Currently, bisphosphonates, calcitonin, and estrogen are often used to treat osteoporosis and related fractures [14,15]. However, these pharmacological treatments have serious side effects, including hypercalcemia, increased risk of breast and endometrial cancer, and gastrointestinal intolerance [16], which may limit their use. Therefore, development of safer and more effective drugs or natural products to prevent osteoclast-triggered bone mass loss is urgently needed. Fucoidan, a fucose-containing sulfated polysaccharide present in brown algae, exhibits several beneficial functions, including anti-inflammatory, antioxidant, anticancer, and immunomodulatory activities [17]. Previous studies have reported that fucoidan inhibits RANKL-induced osteoclast formation in bone marrow-derived macrophages (BMMs) mainly through inhibition of NF-κB activation, a prerequisite for osteoclast differentiation [18,19]. However, the exact mechanisms accounting for the inhibition of osteoclastogenesis by fucoidan remain unclear. In this study, we further investigated the molecular mechanisms by which fucoidan affects osteoclast differentiation and inflammatory bone loss, especially focusing on the alterations in Akt/GSK3β/PTEN/NFATc1 and calcium/calcineurin signaling pathways. 

## 2. Results

### 2.1. Fucoidan Inhibited RANKL-Induced Osteoclast Differentiation and Bone Resorbing Activity

**Figure 1:**
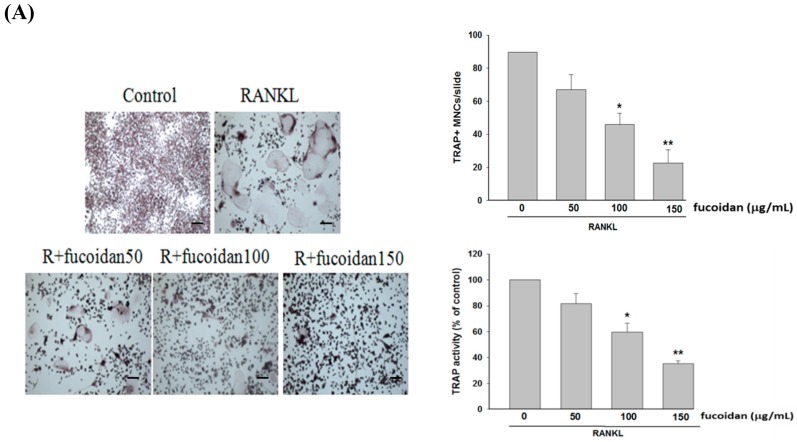

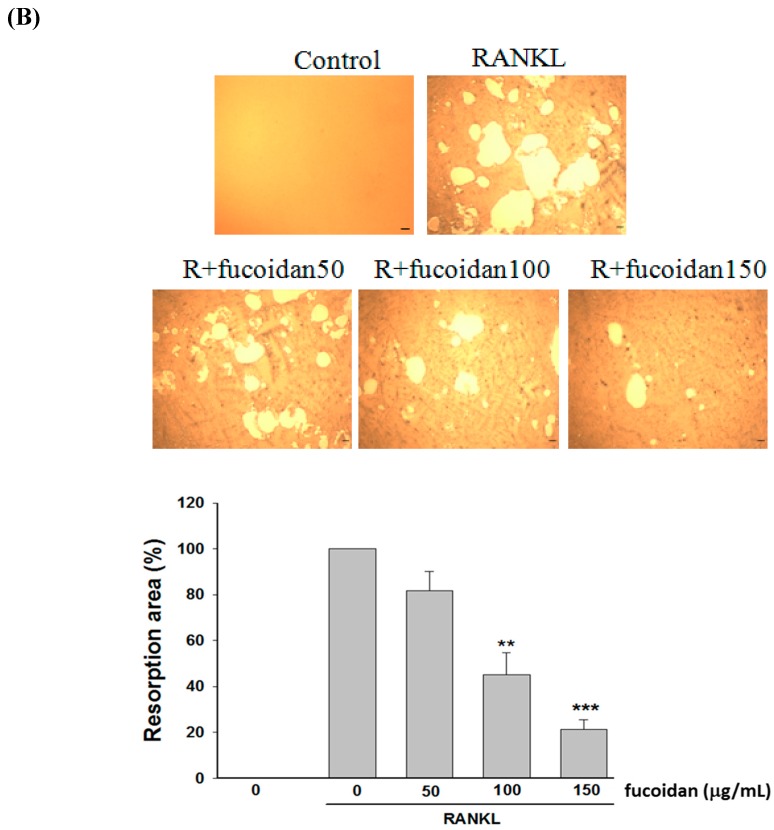
Effects of fucoidan on receptor activator of nuclear factor-κB ligand (RANKL)-induced osteoclast differentiation and bone resorbing activity. RAW 264.7 cells were treated with indicated concentrations of fucoidan in the absence or presence of RANKL (50 ng/mL) for five days. Then, the cells were fixed and stained with TRAP. The TRAP-positive multinucleated cells (TRAP^+^ MNCs) containing three or more nuclei were counted. The TRAP activity was assessed as described in the Method section (**A**). RAW 264.7 cells were incubated with fucoidan (50, 100, or 150 μg/mL) for six days in the absence or presence of RANKL (50 ng/mL). The relative resorption area was expressed as percentage of the values of RANKL-treated alone cells (**B**). (scale bar = 100 μm). Data were expressed as mean ± SD. * *p* < 0.05, ** *p* < 0.01, *** *p* < 0.001 versus RANKL-treated alone cells.

RANKL-induced osteoclast differentiation from RAW 264.7 macrophages accompanied by elevation of TRAP-positive multinuclear osteoclasts and their activities were significantly reduced by fucoidan in a dose-dependent manner (Figure 1A). Consistently, RANKL-induced bone resorbing activity of osteoclasts, which was evaluated by pit formation assay, was markedly inhibited by fucoidan (Figure 1B). These findings indicated that fucoidan has an ability to inhibit RANKL-induced osteoclast formation and bone resorbing activity. After cells were treated with different doses of fucoidan (50, 100, or 150 μg/mL) for 48 h, the cell viability evaluated using 3-[4,5-dimethylthiazol-2-yl]-2,5-diphenyl tetrazolium bromide (MTT) analysis was 92.7 ± 1.2%, 93.4 ± 1.5%, and 93.2 ± 1.3%, respectively, when compared to that of untreated cells. Thus, the effects of fucoidan at the dose range were not due to its cytotoxicity.

### 2.2. Fucoidan Inhibited RANKL-Activated NFATc1 Nucleotranslocation

**Figure 2:**
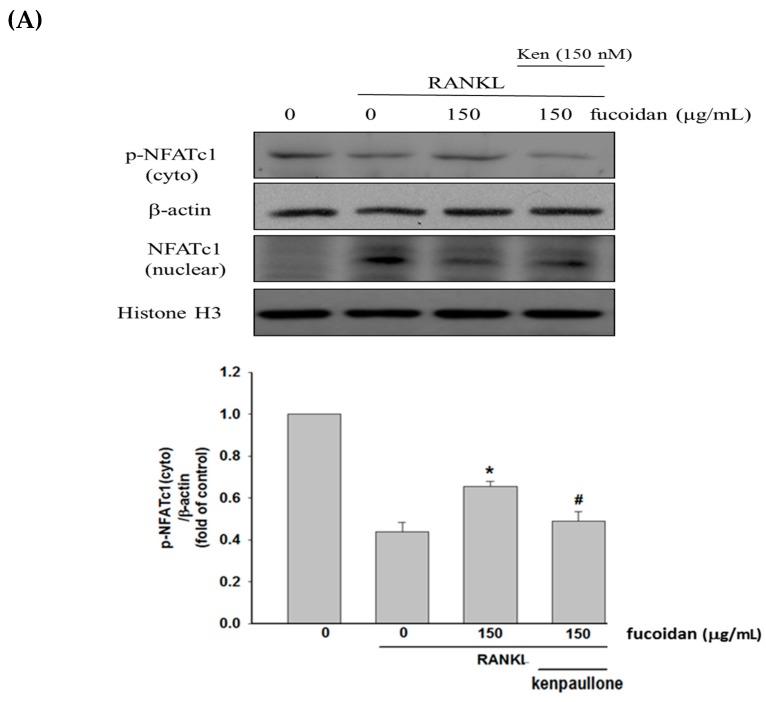

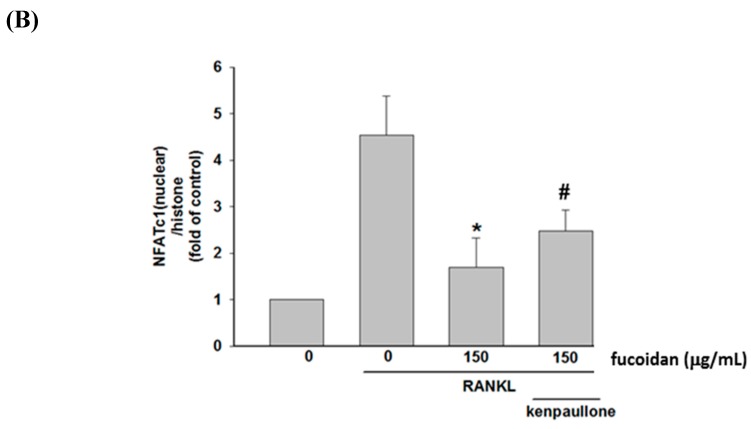
Effects of fucoidan on RANKL-induced NFATc1phosphorylation. Cells were incubated with fucoidan (150 μg/mL) or combination with kenpaullone (Ken, 150 nM) followed by addition of RANKL (50 ng/mL) or vehicle for 24 h. The expression of cytoplasmic p-NFATc1 (**A**) and nuclear NFATc1 expression (**B**) was determined by Western blotting. Data were expressed as mean ± SD. * *p* < 0.05 versus RANKL-treated alone cells. ^#^ *p* < 0.05 versus RANKLand fucoidan group.

Compared to untreated cells, decreased phospho-NFATc1 (inactive form) in the cytoplasm (Figure 2A) and increased nuclear levels of NFATc1 (Figure 2B) were seen in RANKL-treated cells and the events were greatly inhibited by fucoidan. To examine the role of GSK3β, kenpaullone, a GSK3β inhibitor, was added. Our data showed that co-treatment with kenpaullone significantly reversed the actions of fucoidan on NFATc1 phosphorylation and nucleotranslocation. Thus, fucoidan-mediated inhibition of NFATc1 activation is, at least partly, regulated by GSK3β-dependent processes.

### 2.3. Fucoidan Regulated the Akt/GSK3β/PTEN Cascade 

**Figure 3:**
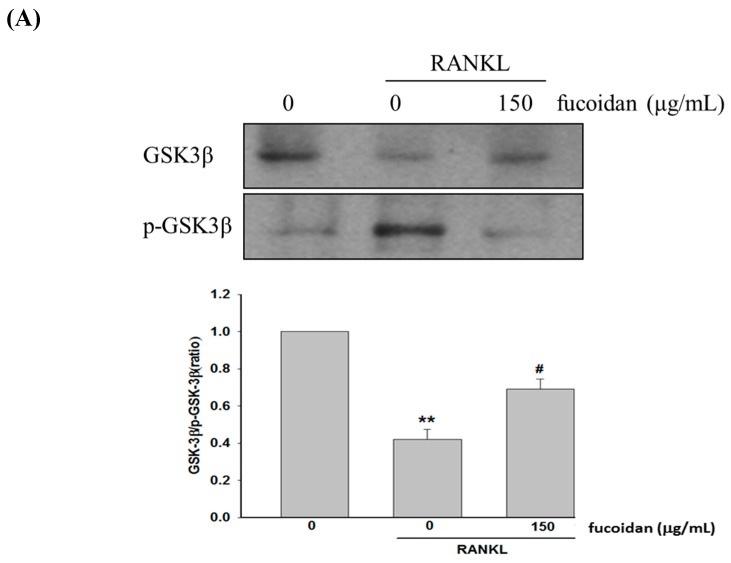

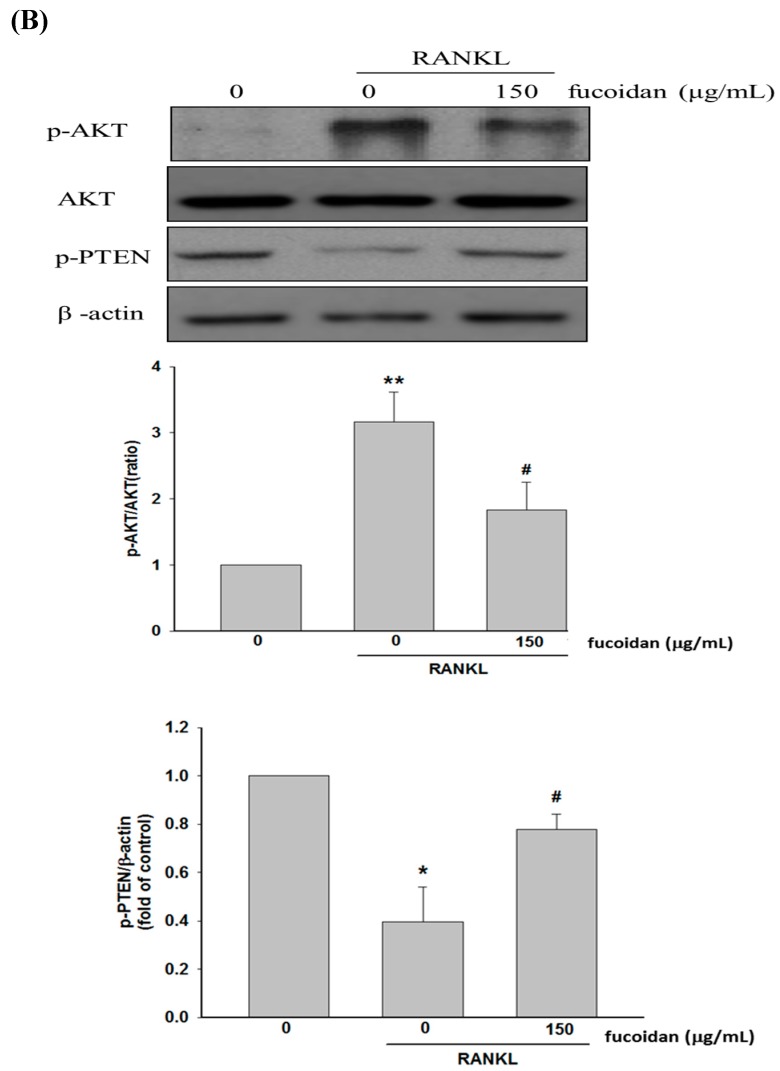
Effects of fucoidan on the expression of GSK3β, p-GSK3β, p-Akt, Akt, and p-PTEN expression. RAW 264.7 cells were pretreated with fucoidan (150 μg/mL) for 2 h followed by addition of RANKL (50 ng/mL) or vehicle for 24 h and the protein expression of GSK3β and p-GSK3β was determined (**A**). After treatment with RANKL or vehicle for 15 min, the expression of p-AKT, Akt, and p-PTEN of various groups was determined (**B**). Data were expressed as mean ± SD. * *p* < 0.05, ** *p* < 0.01 versus untreated cells. ^#^ *p* < 0.05 versus RANKL-treated alone cells.

It is known that activation of Akt results in GSK3β phosphorylation and PTEN inactivation, which ultimately enhances NFATc1 nucleotranslocation and osteoclast differentiation [12,13]. Our data showed that treatment with fucoidan decreased the phosphorylation of GSK3β (Figure 3A) and Akt but increased PTEN phosphorylation (Figure 3B) compared to that of RANKL-treated alone cells. Accordingly, fucoidan-mediated GSK3β activation may result from suppressing Akt phosphorylation due to increased PTEN phosphorylation by GSK3β.

### 2.4. Fucoidan Inhibited RANKL-Evoked [Ca^2+^]i and Calcineurin Activity

**Figure 4:**
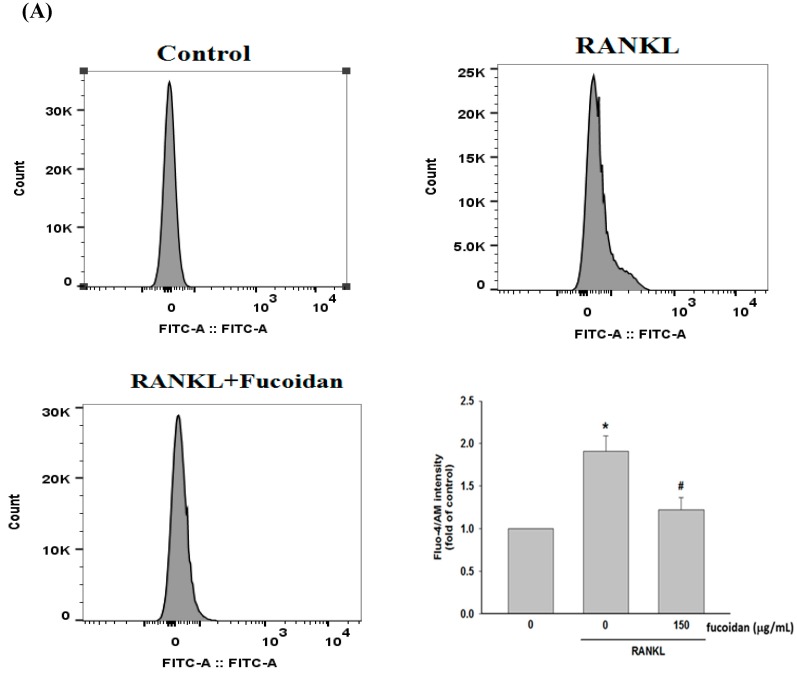

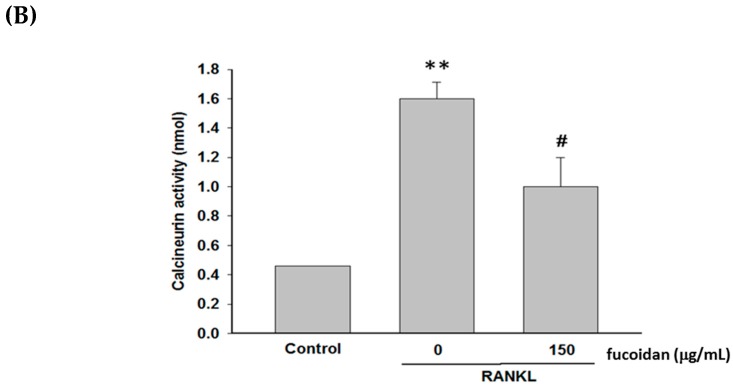
Effects of fucoidan on [Ca^2+^]i and calcineurin activity in RANKL-stimulated RAW 264.7 cells. Cells pretreated with fucoidan (150 μg/mL) for 2 h were incubated with Fluo-4/AM (5 μM) for 30 min followed by stimulation with RANKL (50 ng/mL) for 10 min before the analysis by flow cytometry (**A**). Cells were treated with RANKL for 30 min in the absence or presence of fucoidan, and the cell homogenates were prepared for calcineurin activities assay (**B**). Data were expressed as mean ± SD. * *p* < 0.05, ** *p* < 0.01 versus untreated cells. ^#^ *p* < 0.05 versus RANKL-treated alone cells.

RANKL-stimulated increase in [Ca^2+^]i and calcineurin activity has been considered to enhance NFATc1 nuclear translocation via dephosphorylation of NFATc1 [8]. RANKL-induced increase in [Ca^2+^]i (Figure 4A) and calcineurin activity (Figure 4B) were greatly inhibited by fucoidan. These results suggest that suppressing calcium and calcineruin cascade was an important mechanism contributing to the reduction of NFATc1 activation.

### 2.5. Fucoidan Prevented Lipopolysaccharide (LPS)-Induced Bone Loss in Mice 

**Figure 5:**
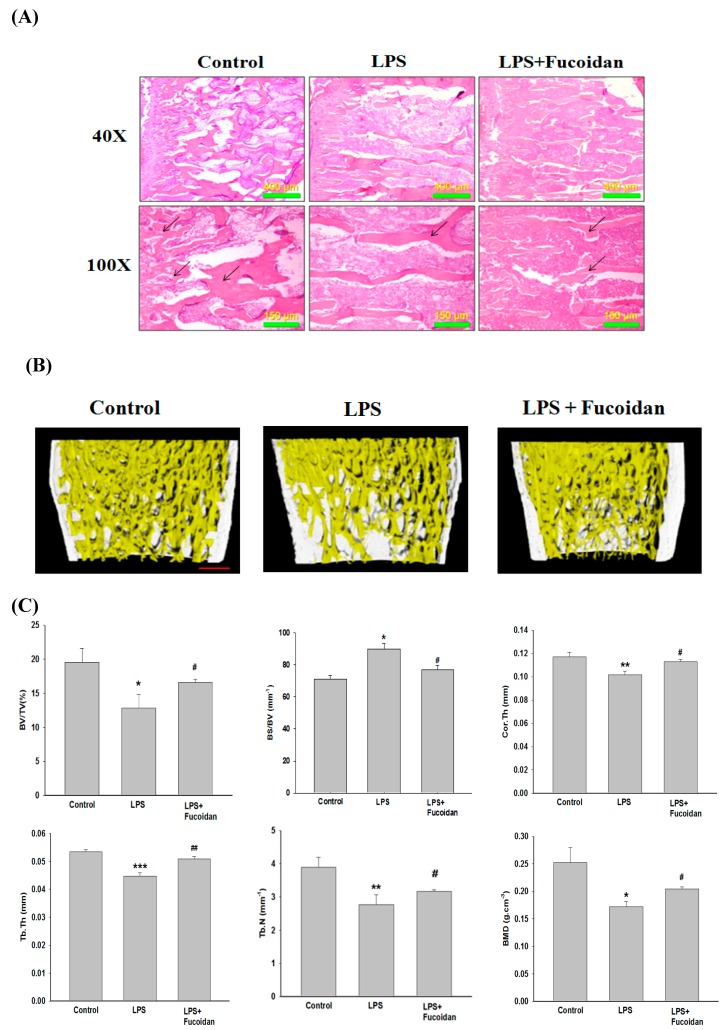
Effects of fucoidan on lipopolysaccharide (LPS)-induced bone loss. Mice were injected intraperitoneally with fucoidan (150 mg/kg body weight) or PBS acting as a control group one day before injection with LPS (5 mg/kg body weight). Then, fucoidan or PBS was injected intraperitoneally every other day for eight days. LPS was injected intraperitoneally on day one and four. All mice were killed eight days after the initial LPS injection. The histological changes of femurs were examined by staining with hematoxylin and eosin (H&E) (40× and 100×) in various groups (scale bar = 150 μm) (**A**). The femurs of various groups were scanned with a high-resolution microcomputer tomography (micro-CT). (**B**) The calculation of the microstructural indices was performed with the micro-CT data (**C**), including bone volume per tissue volume (BV/TV), bone surface/volume ratio (BS/BV), bone mineral density (BMD), average cortical thickness for both cortices (Cor.Th), trabecular separation (Tb.Sp.), trabecular thickness (Tb.Th.), and trabecular number (Tb.N). Data were expressed as mean ± SD. * *p* < 0.05, ** *p* < 0.01, *** *p* < 0.001 versus control group. ^#^ *p* < 0.05, ^##^ *p* < 0.01 versus LPS-injected alone group.

The bone morphometric examination showed that severe bone loss was observed in LPS-treated mice compared to that in control mice (Figure 5A). The images of microcomputer tomography (micro-CT) also confirmed that injection of LPS caused bone loss in mouse femurs reflected by a marked reduction in trabecular bone mass (Figure 5B). The analysis of various parameters, including bone volume/tissue volume (BV/TV), bone surface/volume ratio (BS/BV), bone mineral density (BMD), average cortical thickness for both cortices (Cor.Th), trabecular thickness (Tb.Th.), and trabecular number (Tb.N) used to evaluate the bone loss strongly confirmed that LPS was a potent stimulator of bone loss (Figure 5C). Consistently, treatment with fucoidan (150 mg/kg body weight) significantly reduced the extent of bone loss caused by LPS, indicating that fucoidan was able to prevent inflammatory bone loss in vivo.

## 3. Discussion

The net effects of osteoblastic bone formation and osteoclastic bone resorption determine the amount of bone mass. Inhibition of excessive osteoclast formation and activity has been considered as a major target for reducing osteolytic bone loss [20]. In this study, fucoidan was proven to be a potent inhibitor on RANKL-induced osteoclastogenesis via regulation of Akt/GSK3β/PTEN and calcium/calcineurin cascades, thereby inhibiting NFATc1 activation. In addition, fucoidan treatment was capable of preventing LPS-induced inflammatory bone loss in vivo. 

Our results revealed that increased osteoclast differentiation and bone resorption evidenced by an elevation of TRAP-positive multinucleated osteoclasts, TRAP activity, and Pit formation assay in RANKL-treated macrophages were markedly inhibited by fucoidan. Then, the molecular mechanisms underlying the anti-osteoclastogenic activity of fucoidan were investigated. NFATc1 is a critical transcription factor in promoting RANKL-induced osteoclastogenesis by activating the transcription of genes involved in osteoclast differentiation and activity [21]. RANKL-induced PI3K/Akt activation causing GSK3β phosphorylation and inactivation results in NFATc1 nuclear translocation due to attenuation of NFATc1 phosphorylation [12]. However, GSK3β-dependent phosphorylation of PTEN leads to activation of PTEN, which in turn inhibits PI3K/Akt signaling and activates NFATc1 [13]. Therefore, there is a regulatory loop among Akt, GSK3β, and PTEN. Fucoidan treatment diminished RANKL-induced phosphorylation of Akt and GSK3β but enhanced PTEN phosphorylation, thereby increasing NFATc1 phosphorylation and suppressing translocation of NFATc1 into the nucleus. However, blocking GSK3β with kenpaullone greatly reversed the effects of fucoidan on NFATc1. Accordingly, the antiosteocalstogenic activity of fucoidan may be associated with NFATc1 inactivation in a GSK3β-dependent manner. RANKL also increases intracellular Ca^2+^ concentration by DNAX-activating protein 12, Fc receptor common chain, and phospholipase Cγ, and subsequently activates calcineurin and NFATc1 [22]. Similarly, the increased [Ca^2+^]i and calcineurin activity observed in RANKL-treated RAW264.7 cells were greatly diminished by fucoidan. Collectively, the inhibitory effect of fucoidan on osteoclast differentiation may be mediated by regulation of the PTEN/Akt/GSK3β/NFATc1 signaling pathway and suppression of calcineurin activity. 

The inflammatory bone resorption occurring in rheumatoid arthritis and periodontitis has been a major clinical problem. Under inflammatory conditions, overproduction of proinflammatory cytokines such as interleukin-1 and tumor necrosis factor alpha is crucial to trigger osteoclastogenesis and osteolytic bone resorption [23]. Accumulating evidence has indicated that inflammatory cytokine-promoted osteoclastogenesis and bone resorption may be due to enhancement of RANKL production by osteoblast precursors and mature osteoblasts [24,25] and synergy with RANKL to amplify RANKL/RANK-regulated processes [26]. Moreover, the interaction between immune cells and bone cells including osteoblasts and osteoclasts results in an imbalance in bone metabolism by favoring bone resorption [27]. The LPS, a membrane component of Gram-negative bacteria, can enhance the recruitment of various immune cells such as monocytes and macrophages, which further activates the secretion of pro-osteoclastogenic cytokines [28] and preosteoclast fusion and survival [29] that are essential for osteoclast formation and bone resorption. Thus, LPS has been used in most studies to induce inflammatory bone loss in vivo. Fucoidan has been confirmed to exert an anti-inflammatory effect in LPS-stimulated macrophages by suppressing inflammatory cytokine formation and the expression of mitogen-activated protein kinases [30]. Consistent with the results obtained in the in vitro study, treatment with fucoidan significantly mitigated the severity of bone erosion evidenced by an increase in BV/TV, Tb.Th, BMD, and Cor.Th, and a decrease in BS/BV in micro-CT scanning, as well as the examination of bone morphometry in LPS-induced inflammatory bone loss animal model. It has been demonstrated that LPS promotes osteoclast differentiation and the expression of osteoclast-related genes including TRAP, RANK, and MMP-9 in RAW264.7 cells [31]. Thus, suppression of excessive osteoclastogenesis induced by proinflammatory mediators could be a key target to reduce LPS-induced inflammatory bone loss in vivo. Based on the inhibitory effect of fucoidan on osteoclastogenesis in vitro, we expected that there would be similar results in the murine model of inflammatory bone loss. Due to the limitation of the bone tissues, the TRAP staining and related target gene expression were not examined in the animal study, which may be evaluated in future studies. Notably, fucoidan has been reported to enhance osteoblast differentiation through activation of ERK, JNK, and bone morphogenetic protein 2 (BMP-2)-Smad 1/5/8 signaling [32]. Moreover, fucoidan increases bone density and bone ash weight in mice due to upregulation of BMP-2, collagen I, and osteonectin, as well as the activity of alkaline phosphatase and osteocalcin [33,34] that are associated with bone mineralization and osteogenesis. Therefore, the ability of fucoidan to enhance osteoblast differentiation and function may be also involved in its protective effect against bone loss by reversing the imbalance in bone metabolism. As fucoidan has several beneficial effects, fucoidan has become a widely used food supplement especially in Asian countries. A recent clinical study reported that patients with metastatic colorectal cancer receiving 4 g of fucoidan powder twice a day for six months during chemotherapy had a higher disease control rate compared to that of only chemotherapy-treated patients [35]. Thus, oral administration is a potential route of fucoidan administration in patients.

**Figure 6:**
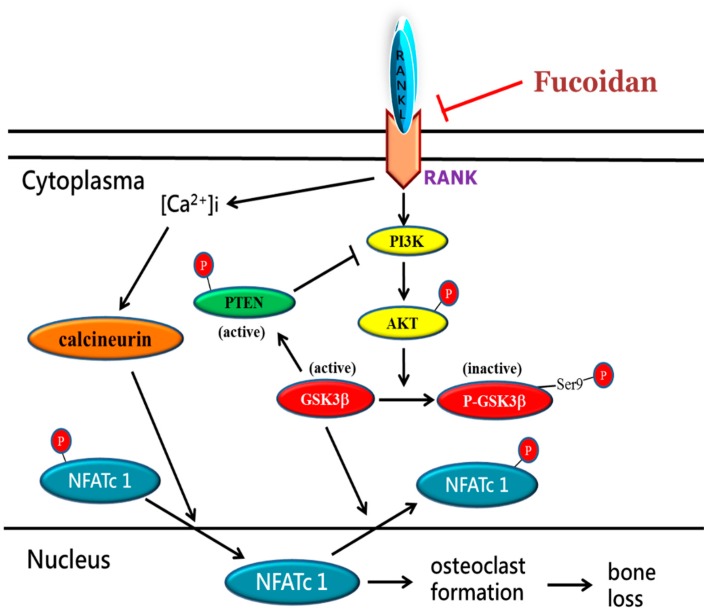
Proposed mechanisms for fucoidan-mediated inhibition of osteoclastogenesis. During RANKL stimulation, fucoidan inhibits Akt phosphorylation leading to GSK3 and PTEN activation, and calcium/calcineurin cascade, which ultimately suppresses nuclear translocation of NFATc1 and osteoclastogenesis-related gene expression, which in turn attenuates osteoclast differentiation.

In summary, we demonstrated that fucoidan inhibits osteoclast differentiation and function in vitro and inflammatory bone loss in vivo. The underlying mechanisms accounting for the anti-osteoclastogenic activity of fucoidan may include suppression of calcium/calcineurin cascade and regulation of the Akt/GSK3β/PTEN/NFATc1 signaling pathway (Figure 6). Taken together, fucoidan could be a potential natural regimen for the treatment of osteoclast-related bone diseases.

## 4. Materials and Methods

### 4.1. Reagents and Fucoidan Preparation 

The antibodies including anti-phospho-GSK3β, anti-GSK3β, and anti-phospho-PTEN were purchased from Cell Signaling Technology (Danvers, MA, USA). The anti-β-actin, anti-NFATc1, and anti-phospho-NFATc1 were purchased from Santa Cruz Biotechnology (Santa Cruz, CA, USA). Other chemical reagents used in this study were analytical grade and obtained from Sigma (Saint Louis, MO, USA). The fucoidan was provided by Hi-Q Marine Biotech International Ltd, Taipei, Taiwan) and prepared as described previously [36]. Briefly, dried *Sargassum hemiphyllum* was incubated with hot water and lyophilized followed by incubation of 95% ethanol for overnight and then glycolytic enzyme was added. The low molecular weight fucoidan with average molecular weight of 800 Da (92.1%) containing fucose 210.9 ± 3.3 μmol/g and sulfate 38.9 ± 0.4% (*w*/*w*) was obtained by passing through suitable molecular weight cut-off membranes. The fucoidan dissolved in distilled H_2_O was used for subsequent tests.

### 4.2. Cell Culture and Cell Viability Assay 

The mouse RAW 264.7 macrophages (Bioresource Collection and Research Center, Hsinchu, Taiwan) were cultured in α-minimal essential medium (α-MEM) (Sigma, St Louis, MO, USA) with 10% fetal bovine serum. Cell viability was examined by the ability of viable cells to reduce MTT to purple formazan, and the absorbance at 570 nm was measured by a spectrophotometer (BioTek, Winooski, VT, USA). 

### 4.3. TRAP Staining and Activity

RAW 264.7 cells were incubated with RANKL (50 ng/mL) and fucoidan for 5 days. The cells were fixed with formaldehyde and washed with PBS at room temperature followed by TRAP staining. TRAP-positive multinucleated cells (MNCs ≥ three nuclei) were counted under a light microscope (Leica, Vertrieb Deutschland, Germany). The cells for TRAP activity assay were treated as described above. After the cells were fixed with formalin and incubated with ethanol/acetone (1:1) for 1 min, the TRAP solution (50 mM citrate buffer (pH 4.6) containing 10 mM tartrate and 5 mM p-nitrophenylphosphate) was added for 1 h. The reaction was stopped with equal volume of 0.1 n NaOH. Then, the absorbance was measured at a wavelength of 410 nm by using a spectrophotometer and the TRAP activity was expressed as a percentage of the activity of RANKL-treated alone cells.

### 4.4. Pit Formation Assay 

RAW 264.7 cells (1 × 105 cells/well) were seeded on Corning® Osteo Assay Surface well (Corning, NY, USA) followed by addition of different concentrations of fucoidan and RANKL (50 ng/mL) for 6 days. Then, cells were removed with 1 n NaOH for 20 min. The resorption pit areas were measured by Metamorph imaging analysis (Metamorph Imaging System, Universal Imaging Corp., Downingtown, PA, USA) and the area was expressed as the percentage of the area of RANKL-treated alone group.

### 4.5. Western Blotting

The nuclear and cytosolic protein was extracted by using NE-PER nuclear and cytoplasmic extraction reagents (Thermo Fisher Scientific Inc., Waltham, UT, USA). The protein samples (20–50 μg) were separated on 8% SDS-PAGE gels and transferred to nitrocelluloss membranes. After blocking with 5% nonfat dry milk in 5% TBST for 1 h, the membrane was incubated with various primary antibody of target genes \ overnight at 4 °C. After washing with TBST three times, the membranes were incubated with a 1:5000 dilution of horseradish peroxidase-conjugated secondary antibody for 1 h at room temperature. The bands were visualized by using the ECL and chemiluminescence reagent (Milipore, Billerica, MA, USA), and the band intensities were quantified with Image J software (LOCI, University of Wisconsin).

### 4.6. Analysis of Intracellular Free Ca^2+^ Oscillation 

RAW 264.7 cells (1 × 10^5^) pretreated with fucoidan for 2 h were incubated with 5 μM Fluo-4/AM (Thermo Fisher Scientific Inc., Waltham, UT, USA), a calcium indicator fluorescent probe, for 30 min at 37 °C in the dark. The fluorescent intensity was measured by using a BD *FACSVerse*™ flow cytometry at an excitation wavelength of 494 nm and an emission wavelength of 516 nm to evaluate the changes of intracellular free Ca^2+^ oscillation. 

### 4.7. Calcineurin Activity Assay

The calcineurin phosphatase activity was measured with a colorimetric calcineurin cellular activity assay kit (Abcam, Cambridge, MA, USA). In brief, cells were lysed using lysis buffer containing protease inhibitors. The protein (5 μg) of cell extracts was used to determine the calcineurin activity by measuring the absorbance at 620 nm and the amount of phosphate released by calcineurin was calculated using a standard curve.

### 4.8. LPS-Induced Bone Erosion in Mouse Femurs

C57BL/6 mice (8 weeks old) were used in this study and had free access to tap water and standard food. There were three groups of five mice each. Mice were injected intraperitoneally with fucoidan (150 mg/kg body weight) or PBS (control group) 1 day before injection of LPS (5 mg/kg body weight, i.p.). Then, fucoidan or PBS was injected every other day for 8 days and LPS was injected on days one and four [37]. At the end of the treatment, the bone tissues of mice were collected for subsequent tests. The animal experiments were approved by an Institutional Animal Care and Use Committee, National Defense Medical Center, Taipei, Taiwan.

### 4.9. Bone Morphometric Analyses 

Mouse femur samples were fixed using 4% paraformaldehyde and the microcomputer tomography (micro-CT) (Bruker Skyscan 1272, Kontich, Belgium) was used to scan samples at 4.6 μm resolution. CT scanning was performed at 70 kVp of voltage, 142 μA of current, 1100 ms of exposure time, and with 0.5 mm aluminum filter. Reconstruction of sections was carried out with GPU-based scanner software (NRecon) (SKYSCAN). For trabecular bone analysis in secondary spongisa, the reconstructed images of distal femur were isolated. Region of interest was defined as trabecular bone area of 1.0–3.0 mm below growth plate (445 slices). In addition, the trabecular bone and cortical bone were automatically isolated using CTAn software (Version 1.15.4.0, Skyscan) provided with the SKYSCAN analysis tool. The alculation of BMD, the microstructural indices of BV/TV, BS/BV, Tb.Th, trabecular number (Tb.N), average cortical thickness for both cortices (Cor.Th), and trabecular space (Tb.Sp) were calculated by using CTAn [37]. The density reference was validated by BMD calibration phantoms (0.25 and 0.75 g/cm^3^ hydroxyapatite). For illustration, CTVox (Version 3.0, Skyscan) (Billerica, MA, USA) was used to provide 3D image. The analysis of cortical bone was carried out by measuring the average cortical thickness for both cortices. Moreover, bone tissues were removed and fixed in 4% PFA (Sigma-Aldrich) for 1 day at 4 °C and were then decalcified in 12% EDTA. The decalcified bones were embedded in paraffin and sectioned for morphometric examination by staining with haematoxylin and eosin (H&E).

### 4.10. Statistical Analysis

The data were expressed as the mean ± SD. The statistical analyses between groups were evaluated by one-way ANOVA with a post hoc Bonferroni test. *P* values less than 0.05 were considered to be a significant difference. All experiments were independently repeated at least five times.
